# The role of coumarin biosynthesis in modulating plant drought responses: insights from *PpBMT* overexpression in *Arabidopsis*

**DOI:** 10.1186/s12870-026-09381-1

**Published:** 2026-06-26

**Authors:** Peipei Wei, Qian Ruan, Qihui Yin, Huifang Chu, Yongchang Cao, Cheng Song, Yiwen Zhang, Jun Dai

**Affiliations:** 1 Engineering Technology Research Center of Plant Cell, West Anhui University, Lu’an, Anhui China; 2 Suqian Traditional Chinese Medicine Hospital, Suqian, Jangsu China; 3 College of Life and Health, West Anhui University, Lu’an, Anhui China

**Keywords:** Bergaptol O-methyltransferase, Coumarin, Drought stress, Negative regulation, Transgenic *Arabidopsis thaliana*

## Abstract

**Background:**

PpBMT encodes the bergaptol O-methyltransferase from the medicinal plant Peucedanum praeruptorum Dunn and belongs to the O-methyltransferase gene family. Although the role of O-methyltransferases (OMTs) in regulating drought stress responses through methylation of various secondary metabolites has been established in numerous plants, the physiological function of PpBMT remains poorly understood, particularly in heterologous systems. Notably, PpBMT has been characterized in vitro as highly specific for bergaptol. Whether it exhibits broader substrate recognition or exerts indirect metabolic effects when overexpressed in a heterologous system lacking the complete furanocoumarin biosynthetic pathway remains an open question.

**Results:**

In this study, PpBMT was overexpressed in Arabidopsis, and drought tolerance was systematically compared with that of wild-type (WT) under PEG-induced drought stress. Under drought stress, transgenic lines showed a more sensitive phenotype than WT plants, with significant reductions in biomass, relative water content (RWC) and chlorophyll content. Their osmotic regulation and antioxidant defense systems were also severely impaired. Moreover, transgenic plants exhibited dysregulated expression of key drought-responsive genes, showing transient early hyperactivation followed by suppression. Notably, while transgenic plants accumulated higher concentrations of coumarins, including scopoletin, isoscopoletin, and isomarmesinin, under both normal and stress conditions, these compounds are structurally distinct from bergapten, the direct catalytic product of PpBMT, suggesting that the observed metabolic changes reflect broader network-level perturbations rather than direct enzymatic conversion.

**Conclusions:**

Our results demonstrate that overexpression of PpBMT substantially altered coumarin metabolic flux in Arabidopsis and negatively regulated plant drought tolerance. This finding underscores that enhancing the accumulation of specific secondary metabolites does not necessarily confer improved stress resistance. Rather, the regulatory context, including stress-inducible, tissue-specific, and feedback-sensitive control of gene expression, is a critical determinant of physiological outcome. Although this study did not directly measure energy-related parameters or carbon allocation, and the sample size was modest, the dataset nevertheless provides valuable insights into the role of PpBMT in coumarin metabolism and highlights the importance of regulatory context in determining whether secondary metabolite accumulation improves or compromises plant stress resistance.

**Supplementary Information:**

The online version contains supplementary material available at https://doi.org/10.1186/s12870-026-09381-1.

## Background

OMTs are enzymes that perform many functions involving methyl transfer [1]. Methylation of various secondary metabolites plays a critical role in plant growth and development, particularly in abiotic stress responses [2]. Plant growth, development, and stress responses have been precisely regulated through the methylation of hormones or signaling molecules [3, 4]. Drought stress is one of the primary abiotic stresses limiting crop growth and productivity [5]. It affects plants through a complicated systemic process at morphological, physiological and molecular levels [6]. When water is scarce, plant metabolism is directly affected, and the photosynthetic system is damaged, leading to reduced chlorophyll content [7]. It also indirectly affects plant growth and changes the root structure [8]. The ability of plants to sense and react to drought stress is vital for their survival and adaptation, and OMTs play a key regulatory role in this process [9, 10]. As a representative medicinal plant in the *Apiaceae* family, *Peucedanum praeruptorum* Dunn has a medicinal history of over 1,500 years in China, mainly due to the presence of its main active ingredient coumarin [11]. The formation and accumulation of coumarin and the biosynthetic pathway involve important core factors with the OMTs [12]. Since the medicinal part of *Peucedanum praeruptorum* Dunn is the root, which is particularly sensitive to water deficiency, the drought stress can profoundly impact the secondary metabolism of coumarins [13, 14]. Therefore, understanding the molecular mechanisms of drought response, especially OMT-mediated regulation of secondary metabolite accumulation, is crucial for improving both stress resistance and medicinal quality of this plant.

OMTs play a critical role in plant stress responses [3, 15]. Methylation of functional groups like phenolic hydroxyl and carboxyl groups influences the formation of various substances, including lignin, flavonoids and melatonin that facilitate plant adaptation to adverse stress conditions, including drought, low temperature and high soil salinity [16–19]. For instance, the homeodomain-leucine zipper I transcription factor (MeHDZ14) in cassava has the potential to regulate the gene of *Manihot esculenta Crantz* caffeic acid 3-O-methyltransferase 1 (*MeCOMT1*) to influence lignin synthesis and modify plant architecture (leaf curling and internode shortening) to reduce water transpiration and enhance drought tolerance [20]. Plants usually tend to activate their antioxidant systems and boost antioxidant enzyme activity to a certain extent in response to drought stress so that normal metabolism will not be disrupted during the period of drought stress [21, 22]. Notably, previous studies have demonstrated that heterologous overexpression of *OMT* genes from other plant species can enhance drought tolerance in transgenic models. For example, overexpression of *OMT* genes from *Ligusticum chuanxiong* and from the tea plant (*Camellia sinensis*) in *Arabidopsis* both improved stress tolerance of the transgenic plants [19, 23]. In those cases, the transgenic lines exhibited enhanced antioxidant enzyme activity and chlorophyll content, accompanied by significantly reduced accumulation of malondialdehyde (MDA) and hydrogen peroxide (H₂O₂). Moreover, roots of the plant would change their growth strategies, alter structures and regulate internal water movement to survive drought stress. In citrus plants, the ABA signaling pathway-related factor PtABF4 (*Poncirus trifoliata* ABA-responsive element binding factor 4) promotes root growth and development through regulation of the *OMT* gene *PtCOMT5* (*Poncirus trifoliata* caffeic acid 3-O-methyltransferase 5) [24]. This also enlarges the root uptake region, bolsters the plant’s ability to retain water and absorb nutrients, facilitating the drought resistance of citrus seedlings.

**Fig. 1:**
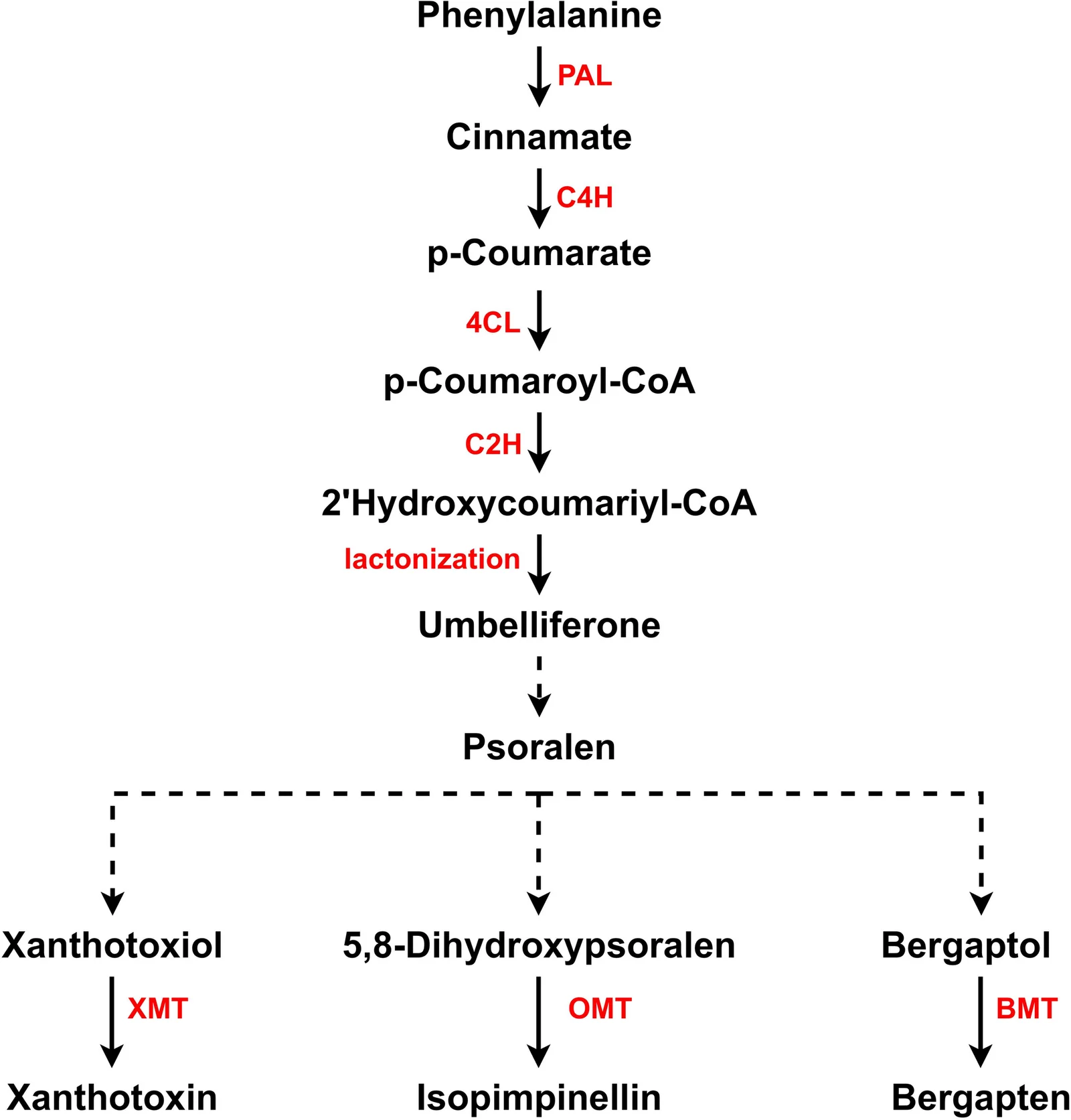
Schematic outline of coumarin biosynthesis in *Peucedanum praeruptorum* Dunn. The dashed line denotes an uncharacterised step whose responsible gene has not yet been cloned

The bergaptol O-methyltransferase (PpBMT) derived from *Peucedanum praeruptorum* Dunn is a specialized class of O-methyltransferase [25]. In its native context, PpBMT displays high substrate specificity for bergaptol, catalyzing the O-methylation of bergaptol to form furanocoumarin compounds like bergapten [26]. This specific catalytic reaction constitutes one of the most critical steps in the biosynthesis of characteristic coumarins in umbelliferous plants (Fig. 1) [27, 28]. However, it should be noted that the substrate range of PpBMT has been characterized primarily through in vitro assays with purified substrates [25, 26]. Whether PpBMT exhibits broader substrate recognition or exerts indirect metabolic effects when overexpressed in a heterologous system that lacks the complete furanocoumarin biosynthetic pathway remains an open question. In the present study, we detected significant alterations in the levels of scopoletin, isoscopoletin, esculetin, and isomarmesinin—simple coumarins that are structurally distinct from bergapten but share upstream biosynthetic origins in the phenylpropanoid pathway (Fig. 1). These changes are unlikely to result from direct enzymatic conversion by PpBMT. Rather, they may reflect broader metabolic network perturbations, as discussed below. Although abiotic stresses such as drought have been shown to precisely induce the expression and activity of OMTs in numerous plant species [29–31], it remains unclear whether PpBMT, a specialized OMT dedicated to coumarin synthesis, possesses a similar stress-responsive function. Our preliminary studies revealed that the *PpBMT* gene exhibits highly significant differential expression in tissues at various developmental stages of *Peucedanum praeruptorum* Dunn [32] and shows strong transcriptional responses to drought stress in the root (Fig. S1). However, the functional role of the *PpBMT* in drought stress, particularly its effect when expressed heterologously, has not been characterized.

Given the contrasting evidence from previous heterologous expression studies and our preliminary expression data, we sought to directly test whether *PpBMT* overexpression is beneficial or not for improving plant resilience to dehydration. To address this question in a controlled heterologous system, we expressed *PpBMT* in *Arabidopsis* and evaluated its impact under drought stress. Our results show that, unlike the positive effects reported for other *OMTs*, *PpBMT* overexpression negatively regulates drought tolerance in transgenic *Arabidopsis*. This is supported by differences in biomass accumulation, chlorophyll content, relative water content, proline, malondialdehyde levels, and antioxidant enzyme activities. The qRT-PCR profiling of drought-related genes under PEG-simulated drought stress, along with these results, clarifies the role of *PpBMT* in plant drought responses. Moreover, transgenic lines that overexpressed *PpBMT* showed significantly higher levels of esculetin, scopoletin, isoscopoletin, and isomarmesinin under drought stress. Together, these findings advance our understanding of how *PpBMT* regulates coumarin metabolism and mediates drought adaptation in a heterologous plant system, while also highlighting that not all *OMTs* confer positive stress tolerance when overexpressed.

## Materials and methods

### Plants and treatments

The experimental materials of *Peucedanum praeruptorum* Dunn were collected from the Medicinal Plant Garden at West Anhui University. In this study, the *Arabidopsis* Columbia ecotype (Col-0) was utilized. Growth conditions were implemented according to the protocol established in our previous study [33]. All plants were grown in a controlled environment chamber under the following conditions: temperature 20 ± 2 °C, photoperiod 12 h light /12 h dark, light intensity 150 µmol·m⁻²·s⁻¹, and relative humidity 70 ± 5%. Five-week-old *Arabidopsis* transgenic overexpression lines (OE) and WT plants (at the growth stage of 8–10 rosette leaves) were used for all experiments. Two independent experimental setups were conducted to accommodate different analytical requirements:

1. Hydroponic system for short-term gene expression analysis (qRT-PCR): Plants were grown in 1/2 Hoagland nutrient solution (pH = 5.8). For drought treatment, 20% (m/v) polyethylene glycol 6000 (PEG-6000) was added to the nutrient solution. Whole plants were collected at 0, 2, 4, 8, 12, and 24 h after PEG addition, immediately frozen in liquid nitrogen, and stored at -80 °C for subsequent RNA extraction and qRT-PCR.
2. Soil culture system for long-term physiological and biochemical assays: Plants were grown in vermiculite. For drought treatment, plants were irrigated with 1/2 Hoagland nutrient solution supplemented with a 20% (m/v) PEG-6000 (treatment group), while control plants were irrigated with the 1/2 Hoagland nutrient solution without PEG-6000 (control group). After about 10 days, when significant phenotypic differences were observed (visible wilting and curled rosette-shaped leaves), seedlings were harvested to measure physiological indices, including biomass accumulation, relative water content, and chlorophyll content, as well as osmotic regulation substances (proline) and a membrane integrity marker (malondialdehyde, MDA). Subsequently, the activities of antioxidant enzymes and changes in coumarin content were analyzed.

### Generation of PpBMT-overexpressing transgenic Arabidopsis.

Following the methodology from our previous study [33], the full-length coding region of the *PpBMT* gene was cloned from *Peucedanum praeruptorum* Dunn (Fig. S2). The *PpBMT* gene (GenBank: KU359196.1) was then inserted into the plant overexpression plasmid pCAMBIA1300 (ABclonal, Changsha, China) using ClonExpress Ultra One Step Cloning Kit V3 (Vazyme, Nanjing, China). The final construct, Pro35S: *PpBMT* recombinant plasmid, was introduced into *Agrobacterium tumefaciens* EHA105, and this strain was used for *Arabidopsis* transformation [34]. Transgenic plants were screened on solid 1/2 MS medium supplemented with 25 µg·mL^− 1^ hygromycin B, followed by molecular identification (Fig. S3). Only homozygous T3 transgenic seeds were used for further analysis.

### Determination of physiological and biochemical parameters

For these measurements, plants from the soil culture system (long‑term treatment) were used. The *Arabidopsis* seedlings were quickly rinsed with clean water to remove vermiculite and residual PEG, then allowed to dry. Three plants from each genotype were randomly selected and their maximum root length was immediately measured, after which the fresh weight (FW) was recorded. Subsequently, the seedlings were placed in deionized water for 24 h to achieve saturation. After recording the saturated fresh weight (TW), the seedlings were subjected to 100 °C for 20 min to terminate biological activity, then dried at 80 °C until a constant weight was achieved, and the dry weight (DW) was recorded. The RWC is calculated using the following formula: RWC(%) = [(FW - DW) / (TW - DW)] × 100%. The activities of ascorbate peroxidase (APX), peroxidase (POD), superoxide dismutase (SOD), and the contents of proline, MDA and chlorophyll in seedlings were measured using detection kits (KTB3091, KTB1150, KTB1030, KTB1430, KTB1050, and KTB3022 Abbkine, Wuhan, China) according to the manufacturer’s instructions.

### Total RNA isolation and real-time quantitative RT-PCR analysis

For gene expression analysis, plants from the hydroponic system (short-term treatment) were used. To investigate the effects of *PpBMT* overexpression on stress-related genes under drought stress, *Arabidopsis* plants were rapidly collected at 0, 2, 4, 8, 12, and 24 h after PEG-6000 treatment. The samples were immediately frozen in liquid nitrogen and stored at -80 °C. Total RNA was extracted from each sample using an RNA extraction kit (9769 S, Takara, Dalian, China), and first-strand cDNA was synthesized using the reverse transcription kit (RR092S, Takara, Dalian, China) according to the manufacturer’s guidelines. qRT-PCR was performed using the TB Green^®^ Fast qPCR Mix (RR430S, Takara, Dalian, China) with an ABI QuantStudio5 Real-Time PCR system (ABI, Q5, USA). The PCR program was set for 5 min at 95 °C, followed by 40 cycles of 5 s at 95 °C, 15 s at 60 °C, and 15 s at 72 °C. The *Arabidopsis AtACT2* (Accession: NM_112764.4)was amplified in parallel as an internal control gene. The relative expression of genes was calculated using the 2^–ΔΔCt^ method [35], and each sample included three biological replicates. All primers used are listed in Table S1.

### Determination of coumarin content

The control group and *Arabidopsis* seedlings subjected to 10-day 20% PEG stress treatment were dried, ground, and passed through a No. 5 sieve. Precisely 0.3 g of the sample was weighed and placed into a stoppered conical flask, followed by the addition of 10 mL of methanol while recording the weight. The mixture was then sonicated at 300 W for 30 min. After allowing the mixture to cool to room temperature, the flask was weighed once more. To offset the weight reduction caused by solvent evaporation, additional methanol was introduced. Following thorough agitation, the supernatant was separated via filtration. The coumarin concentration in the extract was then determined using the ultra-performance liquid chromatography outlined by Liao et al. in their experimental procedures [36].

### Statistical analysis

The experimental data were analyzed with the SPSS 29.0 statistical software. For all datasets except those presented in Fig. 4, Fig. S1 and Fig. S4, each biological replicate consisted of a single individual plant, and three such independent plants were analyzed per genotype and treatment. For Fig. 4, Fig. S1 and Fig. S4, each biological replicate comprised a pool of three individual plants; three independent pools were analyzed per genotype and treatment. All data are presented as mean ± standard deviation (SD) of three biological replicates. Statistical comparisons were performed using Student’s *t*-test. Statistical significance is indicated by asterisks with significance levels denoted as: *p < 0.05, **p < 0.01, ***p < 0.001.

## Results

### Overexpression of the PpBMT reduced biomass and relative water content under drought stress

**Fig. 2:**
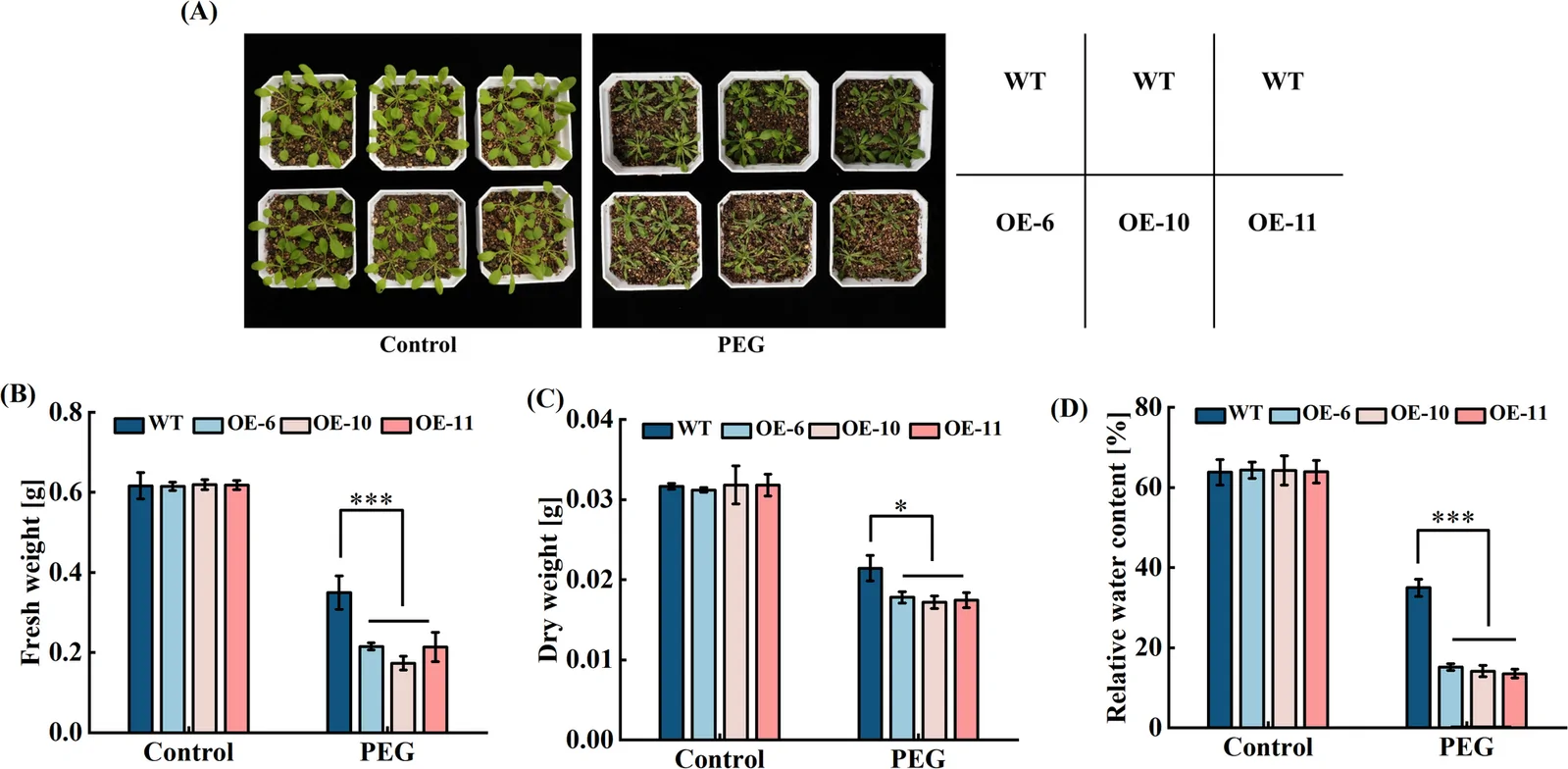
Comparison of physiological parameters associated with PEG‑induced osmotic stress in WT and *PpBMT* transgenic *Arabidopsis* seedlings. (**a**) Growth phenotypes of WT and different *PpBMT* transgenic lines (*OE-6*, *OE-10* and *OE-11*) under control and PEG treatment (**b**) Fresh weight, (**c**) Dry weight and (**d**) RWC were compared between WT and transgenic seedlings with or without PEG treatment. The results were presented as mean ± SD (*n* = 3) of three independent biological replicates, where each replicate represents an individual plant, and significance analysis using Student’s *t*-test (*p < 0.05, ***p < 0.001)

Under normal conditions, no significant phenotypic differences were observed between WT and transgenic *Arabidopsis* (Fig. 2A). Under drought stress, the growth of both was significantly inhibited. However, transgenic plants exhibited a stronger inhibition (leaf curling, wilting). The fresh and dry weights of the transgenic lines were significantly reduced as compared to WT, with maximum reductions of 50.44% and 19.77%, respectively (Fig. 2B and C). And the decrease in fresh weight was particularly pronounced, reaching a highly significant level. In addition, the RWC significantly declined compared to the WT, notably by 61.36% (Fig. 2D).

### Overexpression of the PpBMT decreased chlorophyll content under drought stress

**Fig. 3:**
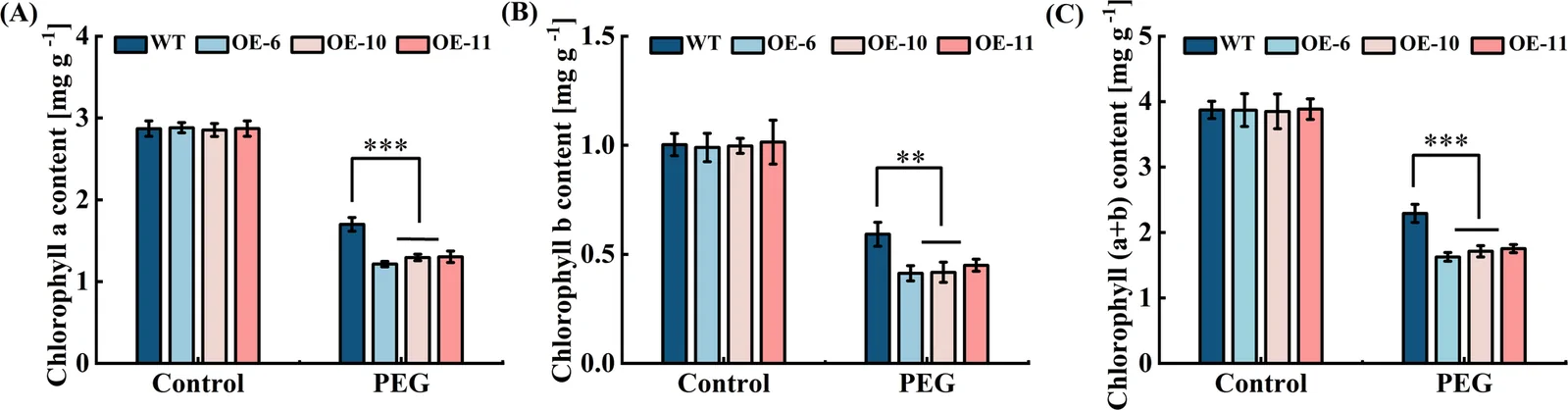
Chlorophyll content in WT and *PpBMT*-transgenic *Arabidopsis* seedlings. (**a**) Chlorophyll a, (**b**) Chlorophyll b, and (**c**) Chlorophyll (a + b) were compared between WT and different *PpBMT* transgenic lines seedlings with or without PEG treatment. The results were presented as mean ± SD (*n* = 3) of three independent biological replicates, where each replicate represents an individual plant, and significance analysis using Student’s *t*-test (**p < 0.01,***p < 0.001)

The content of chlorophyll was no different in the WT and transgenic lines under normal conditions. However, drought stress considerably reduced chlorophyll content in both lines (Fig. 3). Notably, the decline was more dramatic in the transgenic lines. As compared to the WT, the Chlorophyll a showed an average reduction of 34.73% (Fig. 3A) while the chlorophyll b dropped by 27.92% (Fig. 3B). The total chlorophyll content (chlorophyll a + b) was reduced by up to 47.86% in the transgenic lines under drought conditions (Fig. 3C).

### Expression analysis of drought-related genes under drought stress

**Fig. 4:**
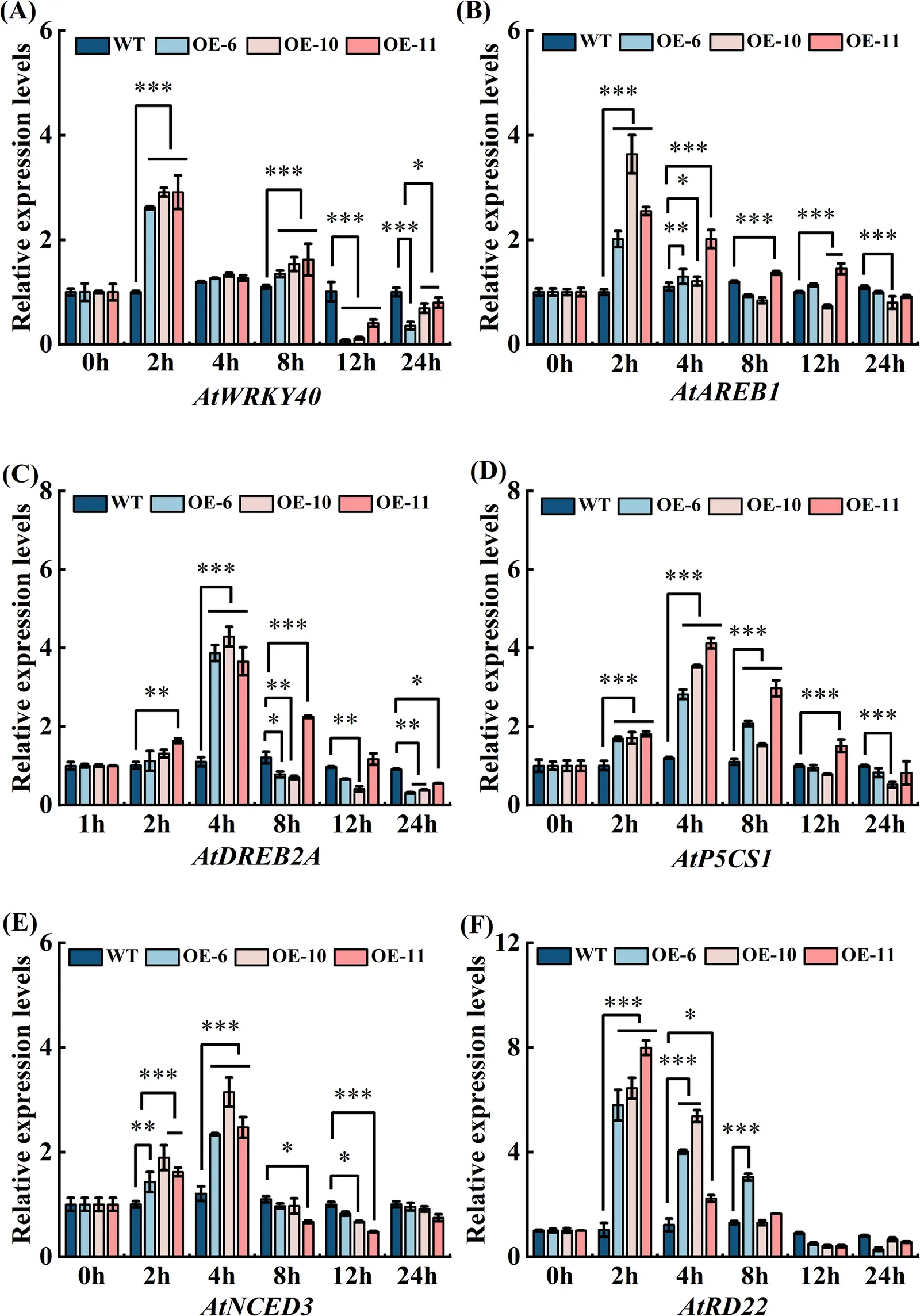
Comparison of drought tolerance gene expression in WT and *PpBMT*-transgenic *Arabidopsis* seedlings. Relative expression of (**a**) *AtWRKY40*, (**b**) *AtAREB1*, (**c**) *AtDREB2A*, (**d**) *AtP5CS1*, (**e**) *AtNCED3* and (**f**) *AtRD22* in *Arabidopsis* seedlings under PEG treatment for 0, 2, 4, 8, 12, and 24 h. Transcript levels were normalized with *AtACT2* (Accession: NM_112764.4). The results were presented as mean ± SD of three independent biological replicates, where each replicate consists of a pool of three individual plants (*n* = 3 pools, each pool from three plants), and significance analysis using Student’s *t*-test (*p < 0.05,**p < 0.01,***p < 0.001)

Under drought stress, the expression levels of drought-related genes in WT changed a little and overall exhibited the expression pattern of an initial increase and later gradual decline (Fig. 4). However, in transgenic *Arabidopsis*, drought-related genes underwent dramatic changes (Fig. 4 and Fig. S4). Although these genes had a similar expression pattern as the WT (first increased, then decreased), the ranges of variation were mostly considerably larger in the transgenic plants. Moreover, the timing of peak expression among those drought-related genes varied across different conditions. The expression of *AtWRKY40*, *AtAREB1* and *AtRD22* peaked at 2 h in the transgenic lines. And the expression levels of these genes were 2.81-fold, 2.73-fold, and 6.58-fold higher than those in the WT (Fig. 4A, B, F), respectively. Genes *AtDREB2A*, *AtP5CS1*, and *AtNCED3* showed peaks at 4 h with an average upregulation of 3.57-fold, 2.91-fold, and 2.2-fold, respectively (Fig. 4C, D, E) with respect to the WT. Surprisingly, the transcript levels of these six genes, which are closely associated with drought response, declined in the transgenic plants despite transient upregulations, and eventually declined to lower levels compared with the WT.

### Overexpression of the PpBMT induced weak activation of the antioxidant enzyme system under drought stress

**Fig. 5:**
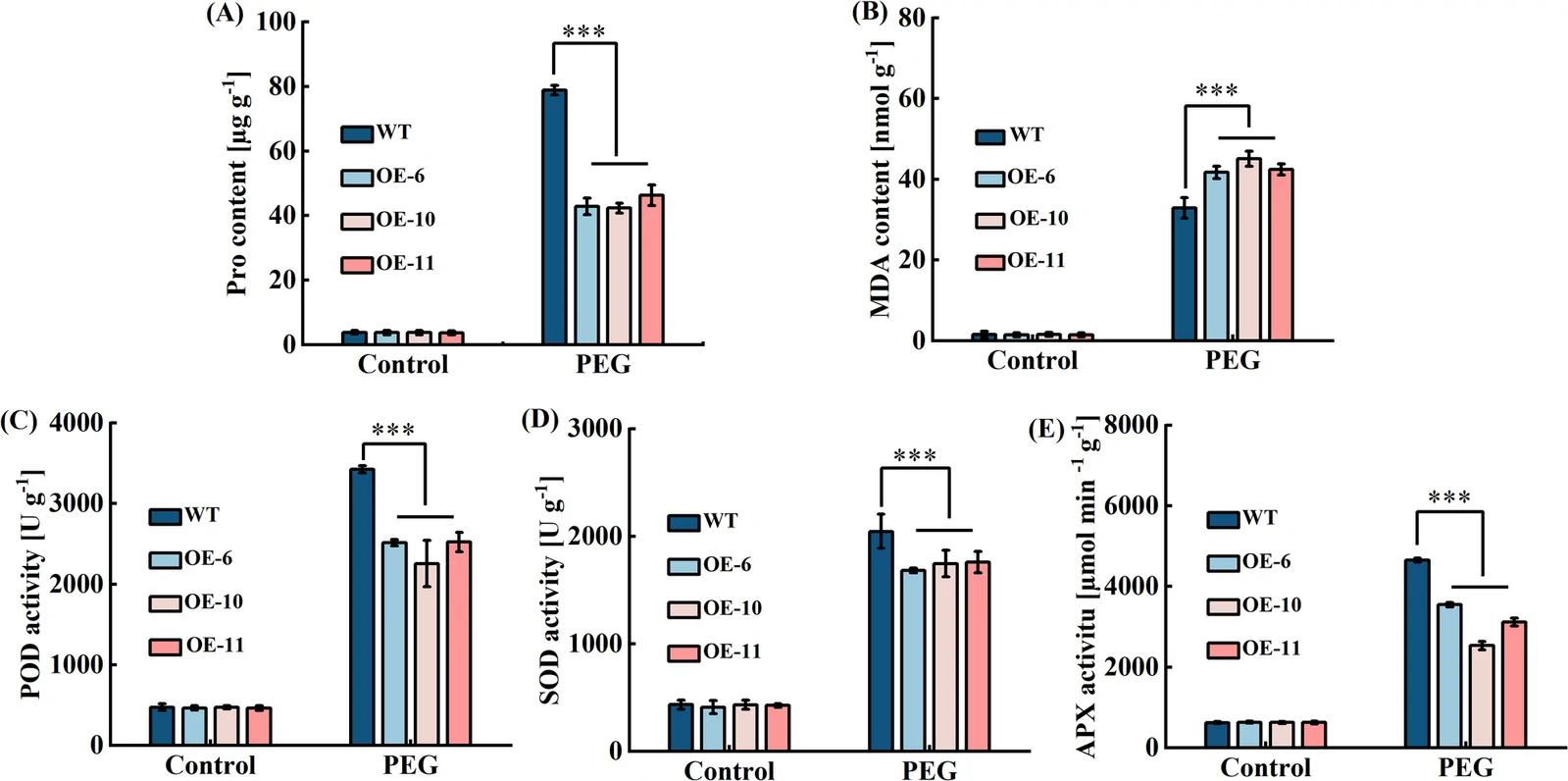
Effects of *PpBMT* overexpression on the osmoregulation and antioxidant system in *Arabidopsis*. (**a**) Pro content, (**b**) MDA content, (**c**) POD activity, (**d**) SOD activity and (**e**) APX activity were compared between WT and different *PpBMT* transgenic lines seedlings with or without PEG treatment. The results were presented as mean ± SD (*n* = 3) of three independent biological replicates, where each replicate represents an individual plant, and significance analysis using Student’s *t*-test (****p < 0.0001)

Under normal conditions, there were no significant differences in proline (Pro), MDA and antioxidant enzyme activity in *Arabidopsis*. Under drought stress, the accumulation of Pro and antioxidant enzyme activity in WT and transgenic plants was both significantly increased, but the increase in transgenic lines was significantly weaker than that in the WT. Compared with transgenic plants, the average increase in Pro content, POD activity, and SOD activity in WT was 79.87% (Fig. 5A), 40.78% (Fig. 5C), and 18.27% (Fig. 5D), respectively. The APX activity is enhanced the most by 83.49% as compared to OE-10 (Fig. 5E). Conversely, MDA levels, as a well‑established stress marker indicating cellular membrane damage, exhibited an inverse pattern: the accumulation in WT was significantly lower than that in transgenic lines by an average of 23.7% (Fig. 5B). This observation is consistent with the other results shown in Fig. 5, as the transgenic lines, which displayed weaker drought tolerance according to the Pro and antioxidant enzyme data, accumulated more MDA under drought stress, reflecting greater oxidative damage.

### Overexpression of PpBMT altered coumarin metabolism in Arabidopsis

**Fig. 6:**
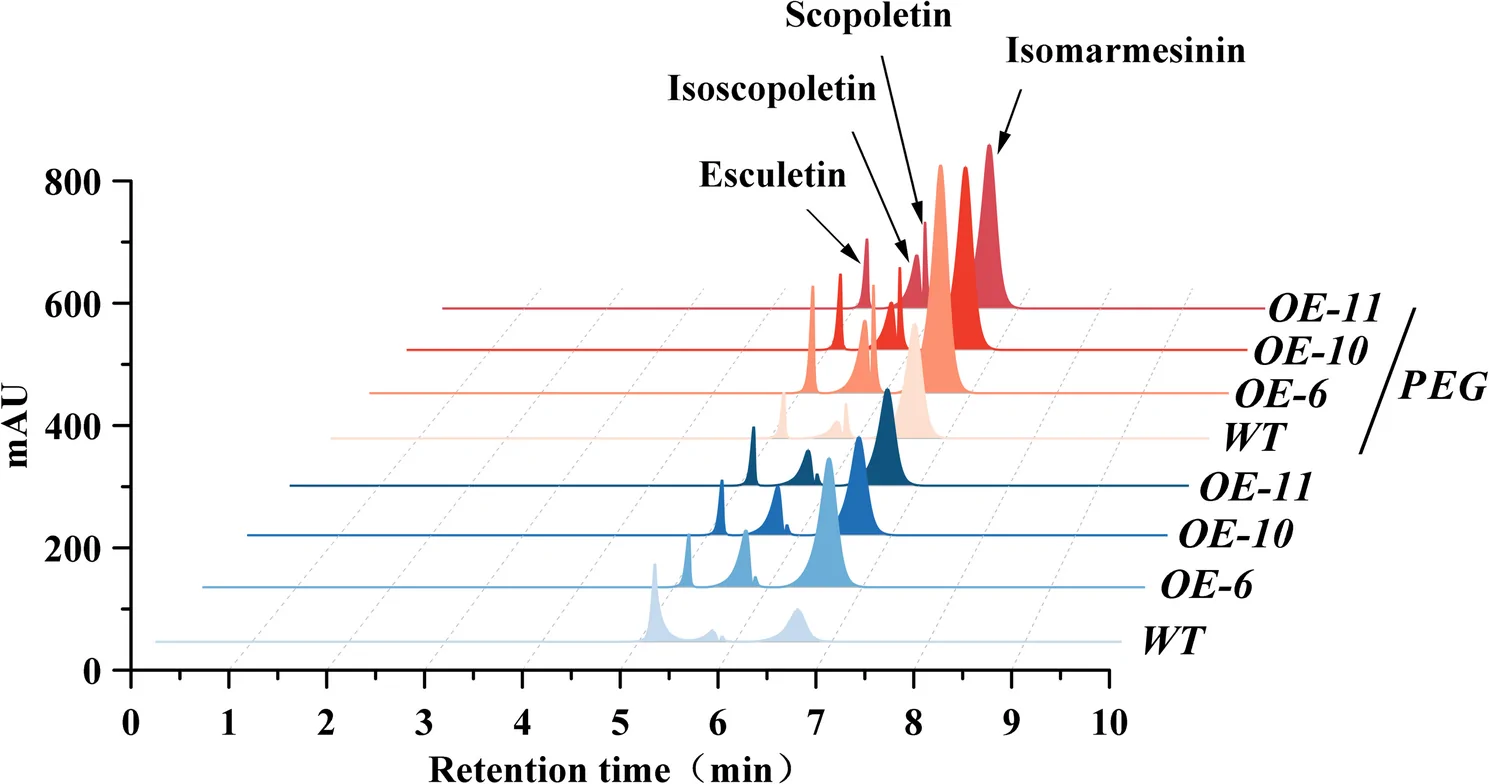
Analysis of coumarins in WT and transgenic *Arabidopsis* by ultra-high performance liquid chromatography. The control was cultured in 1/2 Hoagland solution under normal conditions, while the PEG group underwent PEG stress treatment for 10 days. The Y‑axis indicates absorbance intensity in milliabsorbance units (mAU)

**Fig. 7:**
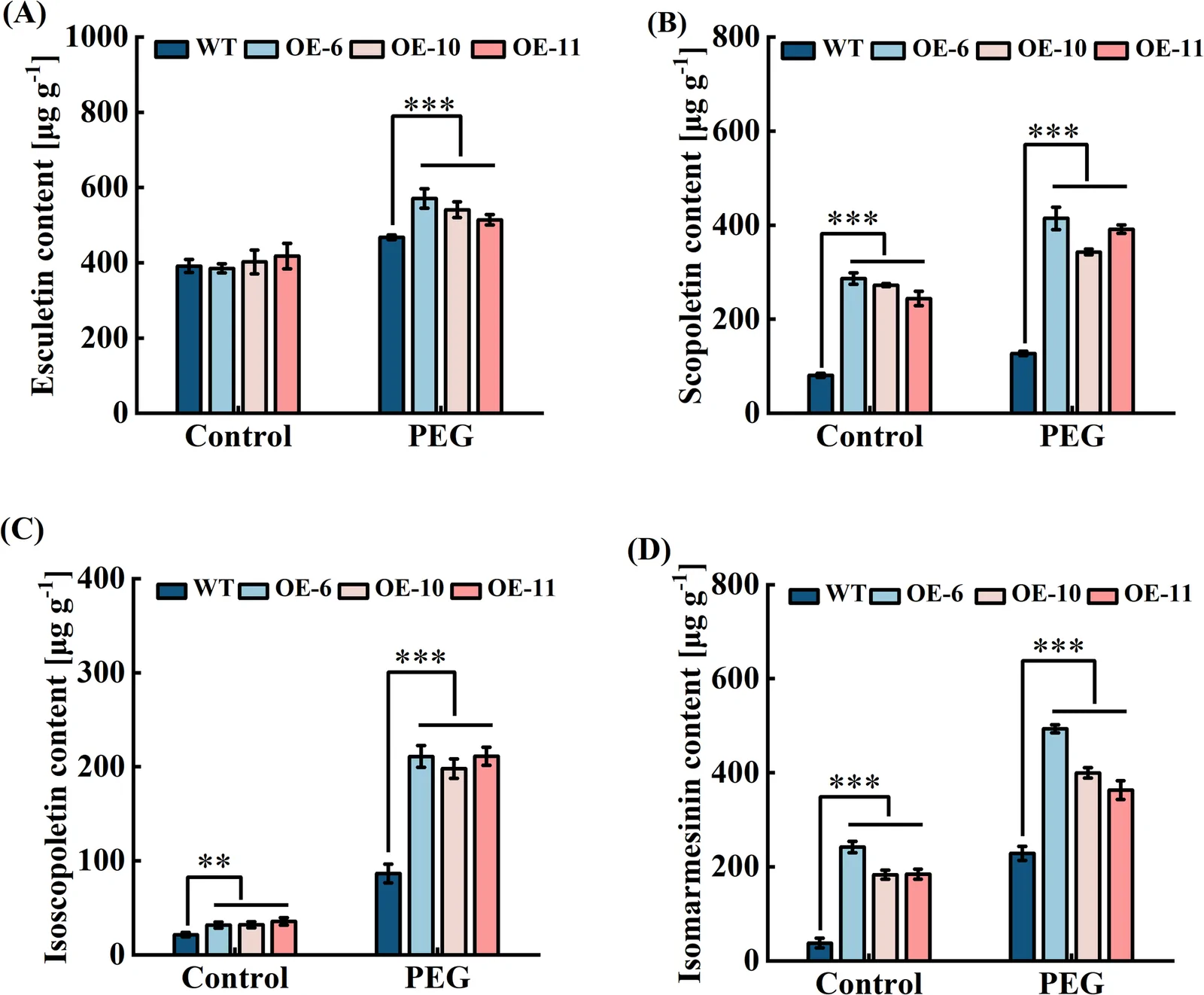
Coumarin contents in WT and *PpBMT*-transgenic *Arabidopsis* seedlings. The contents of (**a**) Esculetin, (**b**) Scopoletin, (**c**) Isoscopoletin and (**d**) Isomarmesinin were compared between WT and transgenic seedlings with or without PEG treatment. The results were presented as mean ± SD (*n* = 3) of three independent biological replicates, where each replicate represents an individual plant, and significance analysis using Student’s *t*-test (**p < 0.01,***p < 0.001)

The metabolic flux of coumarins in *Arabidopsis* was markedly changed by overexpression of the *PpBMT* gene (Fig. 6). Under non-stress conditions (control), transgenic plants already had significantly higher contents of scopoletin, isoscopoletin, and isomarmesinin than the WT, with average increases of 2.32-fold, 0.54-fold, and 4.37-fold, respectively (Fig. 7B-D). Conversely, there was no noticeable increase in the amount of esculetin. The accumulation of these coumarins was further enhanced after drought stress induced by PEG-6000. Notably, the esculetin content of transgenic lines rose by 15.84% in comparison to the WT (Fig. 7A), which is a highly significant difference. However, the transgenic plants maintained significantly higher levels of scopoletin, isoscopoletin, and isomarmesinin than the WT during the course of the treatment.

## Discussion

In this study, we systematically analyzed the effects of *PpBMT* gene overexpression on physiological, biochemical, and molecular responses of *Arabidopsis* under drought stress. Transgenic plants exhibited continuous accumulation of various coumarin derivatives, yet showed greater sensitivity to drought stress. This finding calls into question the prevailing assumption that the accumulation of plant secondary metabolites inevitably enhances stress resistance and instead suggests that the relationship between secondary metabolite accumulation and stress tolerance is highly context-dependent [37].

The *PpBMT* overexpression resulted in remarkable accumulation of scopoletin, isoscopoletin and isomarmesinin in *Arabidopsis*, indicating that PpBMT activity triggers a broader metabolic reprogramming of the coumarin biosynthesis pathway rather than solely affecting its immediate catalytic product (Figs. 6, 7). Despite the enhancement of the metabolic flux, drought resistance did not improve. Instead, phenotypes typical of drought sensitivity, such as diminished biomass accumulation, decreased relative water content, and increased degradation of chlorophyll, were observed (Fig.S2, 3). We propose that one possible explanation for this observed discrepancy is the competitive allocation of energy resources between secondary metabolism and stress adaptation [38]. Plants must divide their resources between growth, primary metabolism, and defence mechanisms under stress [39, 40]. Biosynthesis of coumarins is energy-and carbon-intensive [41, 42], and it is plausible that resources were preferentially diverted toward the synthesis of specific coumarins, most notably isomarmesinin, which accumulated 4.37-fold higher in transgenic lines (Fig. 7D), at the expense of other important processes necessary for drought tolerance. Consistent with this interpretation, both proline accumulation and the activities of antioxidant enzymes (POD, SOD, APX) in transgenic plants were significantly lower than in WT (Fig. 5), and both proline synthesis and the maintenance of antioxidant defense systems are known to be energy-intensive processes [43]. Building on these observations, we hypothesize that overexpression of the *PpBMT* gene in *Arabidopsis* may cause plants to disproportionately allocate metabolic resources toward the synthesis of certain coumarins, and that this shift in carbon and energy partitioning during drought stress may compromise the plant’s ability to maintain cellular water status and redox homeostasis, thereby contributing to the observed decline in drought resistance. We emphasize, however, that this hypothesis remains inferential: direct measurements of ATP levels, adenylate energy charge, NAD(P)H/NAD(P)⁺ ratios, soluble sugar content, and starch reserves would be required to substantiate the proposed trade-off between enhanced carbon flux into coumarin-related metabolism and energy-requiring stress responses. Furthermore, comprehensive metabolomic profiling would help clarify whether the observed coumarin changes arise from direct enzyme activity, metabolic network perturbation, or a combination of both.

Coumarins are a structurally diverse and functionally complex class of compounds [44, 45]. Under the influence of *PpBMT* overexpression, the accumulation of different coumarin components varied significantly. While the accumulation content of esculetin only increased slightly after drought stress (Fig. 7A), that of scopoletin and isomarmesinin remained at a high-level (Fig. 7B, D). This metabolic shift did not enhance drought tolerance in *Arabidopsis* but instead weakened it—an outcome that is contrary to previous findings by Duan et al., who reported that the accumulation of scopoletin enhanced plant drought resistance [46]. However, a closer examination reveals a key distinction: in Duan et al.‘s research, the UDP-glycosyltransferase gene *MaUGT79* is naturally regulated by drought-inducible transcription factors (MaMYB4 and MabHLH11) in its native host (*Melilotus albus*) [46]. This ensures that scopolin (the glucosylated, vacuole-localized form of scopoletin) accumulates only under stress, when it effectively scavenges ROS and reduces oxidative damage. In contrast, our *PpBMT* is driven by a constitutive promoter in *Arabidopsis*, leading to persistent over‑accumulation of free aglycone coumarins without the balancing mechanisms (glycosylation, compartmentalization, stress-responsive regulation) present in the native system. Thus, the apparent discrepancy is not a contradiction but an instructive demonstration that the physiological outcome of coumarin accumulation depends critically on the regulatory context: tight, stress-inducible control and proper metabolic processing can confer benefits, whereas constitutive, unregulated overproduction in a heterologous system may impose a metabolic burden that compromises stress resilience. This resource allocation hypothesis aligns with our observations of reduced antioxidant enzyme activity, lower chlorophyll content, and higher MDA in *PpBMT*-overexpressing lines under drought stress, although we acknowledge that this consistency does not constitute direct proof of causality.

An important question raised by our data is why overexpression of *PpBMT*, an enzyme characterized as bergaptol-specific, leads to pronounced changes in structurally distinct simple coumarins (scopoletin, isoscopoletin, esculetin) and the dihydrofuranocoumarin isomarmesinin, rather than in bergapten itself. We consider several non-mutually exclusive explanations. First, the simple coumarins and furanocoumarins share common upstream precursors in the phenylpropanoid pathway, including umbelliferone, which serves as a key branch-point intermediate [44, 45]. Constitutive overexpression of *PpBMT* may create a metabolic pull on this shared precursor pool, indirectly altering flux through the branches leading to scopoletin, isoscopoletin, and esculetin. Second, although PpBMT has been characterized as highly specific for bergaptol in vitro [25, 26], its substrate recognition properties have not been systematically evaluated in planta, particularly in a heterologous system such as *Arabidopsis*, which does not naturally produce bergaptol. It is conceivable that in the absence of its preferred substrate, *PpBMT* may exhibit relaxed substrate specificity toward structurally related phenolic compounds, although this possibility would require confirmation through in vitro enzyme assays with the relevant coumarin substrates. Third, the observed accumulation of isomarmesinin, a compound structurally more complex than the simple coumarins, suggests that *PpBMT* overexpression may have triggered broader, possibly indirect, reprogramming of endogenous *Arabidopsis* coumarin biosynthetic and modifying enzymes through metabolic crosstalk or feedback regulation. The present study was designed to characterize the physiological consequences of *PpBMT* overexpression rather than to map its full substrate range or delineate the detailed metabolic network responses. Untargeted metabolomic profiling and comprehensive in vitro substrate specificity assays would be required to distinguish among these possibilities and to determine whether the coumarin changes represent direct or indirect consequences of PpBMT activity.

To investigate the underlying mechanisms of *PpBMT*-mediated response to drought stress, we performed a dynamic analysis of the expression of pivotal drought-tolerance genes in *Arabidopsis*. In the earlier stage of drought stress, several core drought stress-responsive genes (such as *AtAREB1*, *AtDREB2A* and *AtNCED3*) in transgenic plants had a transient yet pronounced upregulation (Fig. 4). This early hyperactivation is consistent with the notion that overexpression of *PpBMT* or induced alterations in coumarin metabolism flux may render plants more sensitive to drought signals, potentially leading to an early-stage amplification of the abscisic acid (ABA) signaling pathway [47, 48]. However, this heightened, intense initial response failed to persist, and the expression levels of all these genes declined rapidly during later stages and eventually fell below those of WT (Fig. 4). The pattern of early hyperactivation followed by late-stage decline is suggestive of a dysregulated transcriptional response to drought stress that may involve premature attenuation of signaling capacity [49]. One possibility, consistent with the metabolic trade-off hypothesis outlined above, is that the sustained activation of stress-signaling cascades rapidly depletes the energy or metabolic intermediates required to sustain transcriptionally active defense programs, resulting in an early decline in the expression of stress-responsive genes during the post-stress period [50]. Alternatively, or additionally, a negative feedback regulatory mechanism triggered by the initial hyperactivation may contribute to the subsequent suppression. Distinguishing between these possibilities would require direct measurements of cellular energy status and systematic pharmacological or genetic dissection of the feedback circuits involved. This conceptual framework is entirely consistent with the physiological characteristics that were observed in later stages of stress, including the decrease in proline (Fig. 5A) and antioxidant enzyme activities (Fig. 5C-E) and the increased damage of membrane lipid peroxidation as shown by increased MDA content (Fig. 5B).

The phenomenon revealed in this study shared similar conceptual underpinnings to studies on the regulation of plant cell totipotency [51]. Those studies have shown that a few key genes, such as LEC2 and WRI1, when precisely manipulated, can extensively reprogram the transcriptome of plant cells and activate entirely new developmental programs [52, 53]. Likewise, as a metabolic engineering target, the overexpression of *PpBMT* represents a substantial perturbation of the plant’s endogenous metabolic network. However, this intervention is likely to disturb the delicate, intrinsic balance between the regulatory mechanisms of secondary metabolites and other important biological processes, such as stress response and resource allocation. Our study provides preliminary evidence that focusing solely on increasing flux through a single metabolic pathway, without considering its potential effects on cellular energy status and overall regulatory networks, may lead to unexpected negative phenotypes in heterologous systems. Plant stress resistance is a complex trait regulated by the coordinated action of multiple genes and pathways [54, 55]. Enhancing one node or pathway artificially, such as the productivity of coumarin biosynthesis, may trigger a cascading series of metabolic disruptions that eventually compromise the functioning of the whole stress-adaption system —although we stress that this interpretation remains a conceptual model that our phenotypic and molecular data support only indirectly. Nevertheless, several limitations of the present study should be acknowledged. First, the sample size used for the analysis of growth parameters consisted of only three biological replicates. Although this number is common in exploratory studies, it is relatively low and may not provide sufficient statistical power to reliably detect moderate but biologically meaningful differences in growth parameters among the tested lines under drought stress. Second, and most pertinent to the mechanistic interpretations discussed above, this study focused primarily on phenotypic, biochemical, and molecular responses but did not include direct measurements of several parameters that would be required to validate the proposed mechanisms: (i) energy-related parameters (ATP levels or other indicators of energy metabolism) and (ii) carbon allocation (such as soluble sugars and starch). Consequently, the hypotheses regarding metabolic resource reallocation, energy depletion, and carbon reallocation, although grounded in published literature and consistent with our indirect observations, must be regarded as testable propositions rather than established conclusions. Third, because our study was conducted in a heterologous system (*Arabidopsis*) using a constitutive promoter, the observed negative effects may not fully reflect the endogenous function of *PpBMT* in its native context (*Peucedanum praeruptorum* Dunn), where expression is likely subject to developmental and environmental regulation. Future studies with larger sample sizes and integrated analyses of energy status and carbon partitioning will be necessary to test the metabolic trade‑offs underlying the observed phenotype fully, and inducible or tissue-specific expression systems should be employed to disentangle the effects of gene dosage and regulatory context.

## Conclusion

In this study, constitutive overexpression of *PpBMT* in *Arabidopsis* altered the coumarin metabolic profile and reduced drought tolerance. Our findings demonstrate that the relationship between secondary metabolite accumulation and stress resistance is more nuanced than often assumed: enhancing specific metabolites does not necessarily improve stress tolerance. We propose that the absence of stress-inducible, tissue-specific, or feedback-sensitive regulation of *PpBMT* likely contributed to these negative outcomes, and we outline mechanistic hypotheses, including disproportionate metabolic resource allocation and energy and carbon reallocation, to account for the observed phenotypes. Notably, the detected coumarins are not direct catalytic products of *PpBMT*, suggesting indirect metabolic network effects warranting further investigation. We emphasize that these negative outcomes do not demonstrate that *PpBMT* lacks a beneficial role in stress tolerance. Rather, they underscore the critical importance of regulatory context. While acknowledging the modest sample size and the absence of direct measurements of energy-related parameters and carbon allocation, we believe this dataset provides a valuable foundation for investigating the role of the *BMT* gene family in plant drought resistance and the principles governing metabolic engineering for stress tolerance improvement.

## Supplementary Information

Supplementary Material 1: Table S1 List of primers used in this study. Table S2: the sequence of PpBMT gene used in the study. Fig. S1 Relative expression of the PpBMT gene in the root of Peucedanum praeruptorum Dunn under 20% PEG‑induced osmotic stress. Fig. S2 Cloning of the PpBMT gene in Peucedanum praeruptorum Dunn. Fig. S3 PCR molecular identification of Arabidopsis lines with overexpressed PpBMT genes. Fig. S4 Expression analysis of the PpBMT gene in Arabidopsis under 20% PEG‑induced osmotic stress.

Supplementary Material 2.

## Data Availability

All data supporting the findings of this study are available within the paper and its supplementary information.

## Abbreviations

4CL: 4-coumarate: coenzyme A ligase
APX: Ascorbate peroxidase
BMT: Bergaptol O-methyltransferase
C2'H: Cinnamic acid 2′-hydroxylase
C4H: Cinnamate 4-hydroxylase
OMTs: O-methyl-transferase
PAL: Phenylalanine ammonia lyase
POD: Peroxidase
PRO: Proline
RWC: Relative water content
SOD: Superoxide dismutase
WT: Wild-type
MDA: Malondialdehyde
XMT: Xanthotoxiol O-methyltransferase
