# Supplementary material for: The role of coumarin biosynthesis in modulating plant drought responses: insights from PpBMT overexpression in Arabidopsis
Source: BMC Plant Biol. 2026 Jun 26;26:1577. doi: 10.1186/s12870-026-09381-1 (PMC13573395; doi:10.1186/s12870-026-09381-1)
Supplement: Supplementary file 1 [file 12870_2026_9381_MOESM1_ESM.doc]

**Fig.1** Schematic outline of coumarin biosynthesis in *Peucedanum praeruptorum* Dunn. The dashed line represents the uncertainty that has not yet been cloned.

**Fig.2** Comparison of physiological parameters associated with drought stress in WT and *PpBMT* transgenic Arabidopsis seedlings. (**a**) Growth phenotypes of WT and different *PpBMT* transgenic lines (*OE-6*, *OE-10* and *OE-11*) under different treatments, (**b**) Fresh weight, (**c**) Dry weight and (**d**) RWC were compared between WT and transgenic seedlings with or without PEG treatment. The results were presented as mean ± SD (n=3), and significance analysis using Oneway analysis of variance (ANOVA) and Student’s *t*-test (**p* < 0.05, ****p* < 0.001).

**Fig.3** Chlorophyll content in WT and *PpBMT*-transgenic Arabidopsis seedlings. (**a**) Chlorophyll a, (**b**) Chlorophyll b and, (**c**) Chlorophyll (a+b) were compared between WT and different *PpBMT* transgenic lines seedlings with or without PEG treatment. The results were presented as mean ± SD (n=3), and significance analysis using Oneway analysis of variance (ANOVA) and Student’s *t*-test (***p* < 0.01,****p* < 0.001).

**Fig.4** Comparison of drought tolerance gene expression in WT and *PpBMT*-transgenic Arabidopsis seedlings. Relative expression of (**a**) *AtWRKY40*, (**b**) *AtAREB1*, (**c**) *AtDREB2A*, (**d**) *AtP5CS1*, (**e**) *AtNCED3* and (**f**) in Arabidopsis seedlings under PEG treatment for 0, 2, 4, 8, 12, and 24 h. Transcript levels were normalized with *AtACT2*. The results were presented as mean ± SD (n=3), and significance analysis using Oneway analysis of variance (ANOVA) and Student’s *t*-test (**p* < 0.05,***p* < 0.01,****p* < 0.001).

**Fig.5** Effects of *PpBMT* overexpression on the osmoregulation and antioxidant system in Arabidopsis. (**a**) Pro content, (**b**) MDA content, (**c**) POD activity, (**d**) SOD activity and (**e**) APX activity were compared between WT and different *PpBMT* transgenic lines seedlings with or without PEG treatment. The results were presented as mean ± SD (n=3), and significance analysis using Oneway analysis of variance (ANOVA) and Student’s *t*-test (*****p* < 0.0001).

**Fig.6** Analysis of coumarins in WT and transgenic Arabidopsis by ultra-high performance liquid chromatography. The control was cultured in 1/2 Hoagland solution under normal conditions, while the PEG group underwent PEG stress treatment for 10 days.

**Fig.7** Coumarin contents in WT and *PpBMT*-transgenic Arabidopsis seedlings. The contents of (**a**) Esculetin, (**b**) Scopoletin, (**c**) Isoscopoletin and (**d**) Isomarmesinin were compared between WT and transgenic seedlings with or without PEG treatment. The results were presented as mean ± SD (n=3), and significance analysis using Oneway analysis of variance (ANOVA) and Student’s *t*-test (***p* < 0.01,****p* < 0.001).

**Fig. S1** Relative expression of the *PpBMT* gene in the root of *Peucedanum praeruptorum* Dunn under 20% drought stress. The SAND family protein gene from *Peucedanum praeruptorum*

Dunn was used as the internal reference gene in this experiment.The results were presented as mean ± SD (n=3).

**Fig. S2** Cloning of the *PpBMT* gene in *Peucedanum praeruptorum* Dunn

**Fig. S3** PCR molecular identification of Arabidopsis lines with overexpressed *PpBMT* gene

**Fig. S4** Expression analysis of the *PpBMT* gene in Arabidopsis under PEG treatment. The Arabidopsis *AtACT2* was amplified in parallel as an internal control gene. The results were presented as mean ± SD (n=3).

**Table S1** List of primers used in this study.

| Primer names | Forward primer (5'–3') | Reverse primer (5'–3') | usage |
| --- | --- | --- | --- |
| *PpBMT* | ATGGCAGGAATGAAGACTAGTC | CTACTTGGAAAATTCCATAATCC | RCR |
| InF-*PpBMT* | GGAGAGGACAGGGTACCCGGGGATCCATGGCAGGAATGAAGACT | ACCATGGTACTAGTGTCGACTCTAGACTTGGAAAATTCCATAATCC | In-Fusion Cloning |
| *AtACT2*  *(*Accession: NM_112764.4) | GTCGTACAACCGGTATTGTG | GAGCTGGTCTTTGAGGTTT | qRT-PCR |
| *AtWRKY40*  (Accession: NM_106732.4) | CACTACCCTCGTTGTGAAAG | ATGGCATTGGATGGTTG | qRT-PCR |
| *AtP5CS1*  *(*Accession:*NM_001202786.1)* | GTTTCCTCAGCCGCCGATTTTA | GGAAACGCCACGTGTGGAAC | qRT-PCR |
| *AtRD22*  *(Accession: NM_122472.4 )* | GGTGCCTTTTGGATCGGAGA | AACACCGCGAATGGGTACT | qRT-PCR |
| *AtDREB2A*  *(Accession: AB016570.1)* | CGTTTCAGGATGAGATGTGTGA | CTCATCGTGCATATAAAACGCA | qRT-PCR |
| *AtNCED3*  *(Accession:NM_112304.3)* | CAAGTTCAGATCACTCCCAA | GCTTAAAGCGAAGAGTTCAC | qRT-PCR |
| *AtAREB1*  *(Accession: AB017160.1)* | TGGTGGTCTTGTGGGACTTGG | CCATTACTCTTCCCTATCCCTTC | qRT-PCR |
| *PpBMT* | AAGCCTAGTGAACTTTCCGAC | AAGCACCGTCTTCGTTATTGG | qRT-PCR |
| *PpSAND* | ACAGAAGAGCCTCATGAATCCC | CAAGCAAAGGCGTCATATCAAA | qRT-PCR |

**Table S2:** the sequence of *PpBMT* gene used in the study.

*PpBMT* (1083 bp)

ATGGCAGGAATGAAGACTAGTCCATCTCAAGACGAAGAAGCTTGTGTGCTTGCCATTCAATTAGCAACCTCTACAGTGCTTCCCATGATTCTCAAATCAGCAATAGAGCTTGACATACTAAATACCATTTCCAAAGCTGGCCCCGGTAACTATTTAAGTCCTTGTGATCTAGCCTCTAAGCTTCTCATGTCGAACCCCCATGCACCCATCATGCTTGAACGCATCCTCCGAGTCCTGGCTACCTACAAAGTTCTTGGTTGTAAGCGTAGTGAACTTTCCAACGGTGAAGTTGAGTGGCTCTACTGCTGGACACCAGTGTGCAAGTTCTTGTCCAATAACGAAGACGGGGCTTCTATAGCACCGCTTTTGTTAGTGCACCAGGACAAAGTTCCAATGAAGAGTTGGTATCATGTAACAGATGCAGTTCTTGATGGAGGAACTGCATTTGACAAGGCCTATGGAATGAATATATTTGAATATGCTAGCCAAGATCCTCAATTCAACAAGGTGTTTAATAGGTCAATGGCAGGTCATTCTACTATAACCATGAAAAAAATCCTTGAAACTTACAATGGTTTCGAAGGTCTCAAATCTATAGTTGATGTCGGTGGTGGCAGCGGTGCTACCCTTAATATGATCATCTCCAAGTACCCTACTATTAAAGGCATTAACTTCGACCTTCCTCACGTTGTCGGAGACTCTCCAATTCATCCTGGTGTGGAACATGTAGGGGGAGACATGTTCGCTAGTGTGCCAAAAGGAGATGCCATATTCTTGAAGGTTTGGATATTTCATAGTTGGAGTGATGAAGATTGCCTGAAGATCTTGAAAAATTGTTGCGAAGCTTTGGCAGACAATAAGAAGGTCATCGTTGCAGAATTCATCATTCCTGAAGTTCCGGGTGGTAGCGACGATGCAACCAAGAGTGTGGTTCATCTTGATGCTATCATGTTGGCATATGTTCCAGGGGGAAAAGAGAGGACAGAAAAAGAGTTTGAATCTTTAGCTACAAGAGCAGGATTTAAAAGTTTCCGCAAGGTGTGCTGCGCTTTCAATACTTGGATTATGGAATTTTCCAAGTAG
